# Moderate Chili Consumption During Pregnancy Is Associated with a Low Risk of Gestational Diabetes (GDM) †

**DOI:** 10.3390/nu17061025

**Published:** 2025-03-14

**Authors:** Xiaozhong Wen, Fatima Makama, Ryan Buzby, Jeremy Nguyen, Rose Durnell, Iyobosa Ekhator, Daren Chan, Todd C. Rideout

**Affiliations:** 1 Division of Behavioral Medicine, Department of Pediatrics, Jacobs School of Medicine and Biomedical Sciences, State University of New York at Buffalo, Buffalo, NY 14214, USA; fbmakama@buffalo.edu (F.M.); rosedurn@buffalo.edu (R.D.); darencha@buffalo.edu (D.C.); 2 Department of Biological Sciences, College of Arts and Sciences, State University of New York at Buffalo, Buffalo, NY 14260, USA; ryanbuzb@buffalo.edu (R.B.); iyobosae@buffalo.edu (I.E.); 3 Department of Epidemiology and Environmental Health, School of Public Health and Health Professions, State University of New York at Buffalo, Buffalo, NY 14214, USA; jeremyng@buffalo.edu; 4 Department of Exercise and Nutrition Sciences, School of Public Health and Health Professions, State University of New York at Buffalo, Buffalo, NY 14214, USA; rideout@buffalo.edu

**Keywords:** bean, chili, pulse, legume, diet, dietary intervention, pregnancy nutrition, maternal, gestational diabetes, glucose

## Abstract

**Background/Objectives:** We examined the association between bean consumption and the risk of gestational diabetes mellitus (GDM). **Methods**: We analyzed data from 1397 U.S. pregnant women from Infant Feeding Practices Study II. By using a Diet History Questionnaire, pregnant women were asked about the frequency of consumption and portion size of dried beans, chili, and bean soup over the previous month. They also reported the status of GDM. We used multivariable logistic regression models to examine associations between maternal bean consumption and the risk of GDM, adjusting for socio-demographic and pregnancy-related confounders. **Results**: Mean bean consumption was low among pregnant women: 0.31 cups/week of dried beans, 0.16 cups/week of chili, and 0.10 cups/week of bean soup. Dried bean consumption was relatively high in Hispanic mothers (mean, 0.65 cups/week) and mothers from the East South Central region (0.44). Chili consumption was relatively high in mothers who were Black (0.33), who did not attend college (0.18), who had a household size of 4+ (0.19), whose household income was <USD 25,000/year (0.20), who were WIC recipients (0.18), or who lived in the East South Central region (0.26). Pregnant women who consumed chili one time per month had a lower risk of GDM, compared with never consumers (3.5% vs. 7.4%; confounder-adjusted odds ratio or OR, 0.37 [95% confidence interval or CI, 0.17–0.79]; *p* = 0.011). However, there was no significant association between dried bean (6.6% for one time per week or more vs. 7.0% for never; confounder-adjusted OR, 0.82 [95% CI, 0.41–1.62]; *p*-value = 0.569) or bean soup (4.9% for two–three times per month or more vs. 6.6% for never; confounder-adjusted OR, 0.40 [95% CI, 0.05–3.08]; *p*-value = 0.382) consumption and GDM. **Conclusions**: Bean consumption during pregnancy is low and varies by socio-demographics in the U.S. A moderate frequency of chili consumption may offer some protection against GDM. Replication is needed in larger cohorts with more diverse populations, detailed measures of bean consumption, gold standards of GDM diagnosis, and experimental design. Research in this field can potentially inform dietary approaches to addressing GDM and related morbidities.

## 1. Introduction

Gestational diabetes mellitus (GDM) is a condition during pregnancy characterized by abnormally high maternal blood glucose due to insulin resistance and/or insufficient insulin secretion [1,2]. The prevalence of GDM in the United States has been increasing rapidly recently, from 6.0% in 2016 to 7.8% in 2020 [3]. Risk factors for GDM include unhealthy diet, sedentary lifestyle, pre-pregnancy obesity, advanced maternal age, Hispanic ethnicity, previous history of GDM, a family history of type 2 diabetes, cigarette smoking, and lockdowns due to public emergencies, such as the COVID-19 pandemic [4,5,6,7,8]. Untreated GDM may lead to various delivery and birth complications, including the need for cesarean section delivery, macrosomia, premature birth, and neonatal hypoglycemia [9]. In addition, a meta-analysis demonstrated that mothers with a history of GDM had a 7.43-fold higher risk of developing type 2 diabetes in later life after delivery compared with those who had normoglycemic pregnancies [10].

The typical American diet is high in sodium, saturated fats, and simple carbohydrates [11] and low in fruits, vegetables, and fiber [12]. Previous research suggests that the nutrient intake of pregnant women also reflects this pattern [13,14]. Maternal diet quality can potentially be improved by incorporating more beans, as beans are a nutrient-dense food containing complex carbohydrates, fiber, protein, and an array of micronutrients [15]. Currently, the U.S. Department of Agriculture (USDA) recommends that adults consume 1.5 cups/week of dietary pulses, including beans, lentils, and peas [16]. However, bean consumption amongst the general U.S. population is relatively low, with the average intake being 0.15 cups/day (equivalent to 1.05 cups/week) [17]. In addition, there is substantial variation in bean consumption by socio-demographics among the general population. For example, Hispanics [15] and individuals on lower income [18] are more likely to consume beans. Possible contributors to the variation in bean consumption include culture, accessibility, cooking skills, liking the bean taste, and health knowledge [17]. However, there is little research on the consumption of beans during pregnancy.

In the general population, the consumption of dried beans and other pulses has been linked to increased satiety, healthy body weight regulation, and improved glycemic control [19]. For the latter, several biomarkers of glycemic control (i.e., glycated hemoglobin, fasting blood glucose, fasting insulin levels, etc.) have been found to improve following bean/pulse/legume consumption or the dietary replacement of a common food group (e.g., red meat and white wheat bread) with pulses [20,21,22,23]. These metabolic benefits may be attributed to several pulse-derived nutrients, including fiber (e.g., resistant starch), phenolic compounds, and protein. For example, beans contain 18–34 g of fiber per 100 g of dry matter [24,25,26] and also contain indigestible ‘resistant’ starch, which has been shown to lower the postprandial incremental area under the glycemic response curve (iAUC) [23,27]. Beans, especially darker-colored varieties (e.g., black, red, pink, and brown), are also high in phenolic compounds (e.g., anthocyanins and other flavonoids) [28], which are linked to improved glycemic markers [29].

Large cohort studies have illustrated an association between beans and reduced risk of developing type 2 diabetes [30,31,32]. Research has suggested that the consumption of beans increases the amount of dietary fiber intake, which may lead to a decreased risk of GDM [33]. A previous study reported that consumption of a Mediterranean diet (high in legumes, breads, cereals, vegetables, fruits, fish, olive oil, reduced animal fat, meat, and eggs) led to a 15–38% reduction in the risk of GDM [34]. Similar inverse associations with the risk of GDM were observed for Dietary Approaches to Stop Hypertension (DASH) and the alternate Healthy Eating Index (aHEI), which also involve high consumption of legumes [35]. Further, another study found that the consumption of some specific legumes, such as soy foods and nuts, was inversely associated with the risk of GDM during early pregnancy [36].

However, the independent impact of bean consumption on the development of GDM is understudied, especially in the U.S. pregnant population. The only study that we are aware of reported that compared with mothers who consumed ≤ 1.5 servings of legumes (including beans) per week, mothers who consumed at least 3.3 servings of legumes per week had lower odds of GDM (confounder-adjusted odds ratio [aOR], 0.33 [95% CI, 0.16–0.66]), but those who consumed 1.6–3.2 servings per week did not have significantly different odds of GDM (aOR, 0.79 [0.45–1.39]) [37]. However, this study had some limitations. First, it was conducted among pregnant women from one city (Tehran) in Iran, which might limit the generalizability of results to broader populations, especially in the U.S. and other developed countries. Secondly, the investigators did not distinguish among the various preparation methods (e.g., boiled, fried, and steamed) for beans. These preparation methods may potentially impact the glycemic control properties of bean foods and hence GDM risk by influencing the physiochemical properties of beans (i.e., starch structure) or through the interaction of beans with other food components in mixed dishes (i.e., fat, capsaicin, salt, or other ingredients in chili or bean soup).

Therefore, we aimed to fill the aforementioned research gaps by examining the association of bean consumption with the risk of GDM in a U.S. national pre-birth cohort. With rich data, we were able to distinguish among specific bean foods (dried bean, chili, and bean soup) and include the frequency, portion size, and amount of bean consumption, which, all together, could offer comprehensive knowledge on this topic.

## 2. Materials and Methods

### 2.1. Participants and Setting

We performed a secondary data analysis of 1397 U.S. pregnant women from Infant Feeding Practices Study II (IFPS II). IFPS II was a longitudinal pre-birth cohort study (2005–2007) run by the U.S. Food and Drug Administration (FDA) in collaboration with the Centers for Disease Control and Prevention (CDC). Methodological details on the study population, recruitment, data collection, and follow-up strategies can be found in previous publications [38]. Briefly, this national consumer-based research study followed mothers from late pregnancy through their infant’s first year of life. Data were collected mostly by mailed questionnaires and telephone interviews. Mothers provided information on various topics, including maternal socio-demographics, diet, intrapartum experience, and health, as well as infant feeding, sleep, food allergy, and health.

**Figure 1:**
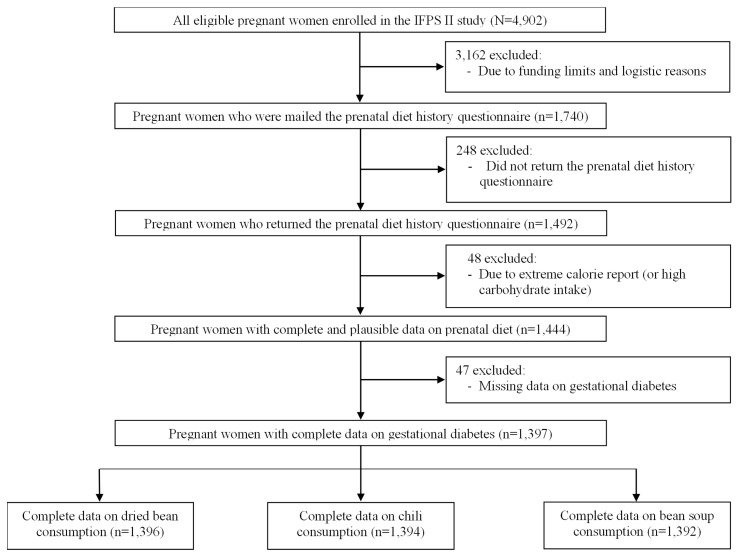
Sample flow chart.

The original full IFPS II study sample consisted of 4902 eligible pregnant women [38]. For this analysis, we restricted the eligible sample to 1397 mothers with complete data on (1) bean consumption during pregnancy (key exposure) and (2) the status of GDM (outcome). Figure 1 shows the sample flow chart. Overall, the distributions of socio-demographic characteristics were considerably different between the analytic sample (N = 1397) and the excluded sample (N = 3505) (Table A1). Specifically, mothers in the analytic sample were more likely to be non-Hispanic White (83.8% vs. 80.2%, *p*-value = 0.016), unemployed (35.7% vs. 32.7%, *p*-value = 0.046), and non-recipients of the Special Supplemental Nutrition Program for Women, Infants, and Children (WIC) (58.9% vs. 52.4%, *p*-value < 0.001); they also had an older mean age (28.8 vs. 27.9 years, *p*-value < 0.001), a higher education level (78.8% vs. 73.8% with college education, *p*-value < 0.001), and higher mean household income (257.5% vs. 243.9% of federal poverty level, *p*-value = 0.025). The IFPS II study materials and procedures were approved by the FDA’s Research Involving Human Subjects Committee (the institutional review board) and the U.S. Office of Management and Budget. Mothers signed informed consent for voluntary participation [38]. The CDC provided our research team access to the de-identified and public-use data of IFPS II. This secondary data analysis was approved by the University at Buffalo Institutional Review Board.

### 2.2. Exposure Measures

In IFPS II, mothers used a modified Diet History Questionnaire (DHQ) to report their dietary intake in the previous month [38]. The original DHQ was a food frequency questionnaire developed and validated by the National Cancer Institute [39]. To fit the pregnant study population, the IFPS II team made some necessary modifications, including replacing the time frame of 1 year with 1 month and adding specific foods relevant to pregnant mothers, such as some types of fish and dietary supplements [38].

For the purpose of this analysis, pregnant mothers reported the frequency of dried bean, chili, and bean soup consumption. For dried bean consumption, mothers were asked, “*Over the past month, how often did you eat cooked, dried beans (such as baked beans, pintos, kidney, blackeyed peas, lima, lentils, soybeans, or refried beans)? (Please don’t include bean soups or chili.)”.* Initially, answer choices included never, 1 time per month, 2–3 times per month, 1 time per week, 2 times per week, 3–4 times per week, 5–6 times per week, 1 time per day, and 2 or more times per day. To ensure sufficient statistical power for analysis, we regrouped answer choices to never, 1 time per month, 2–3 times per month, and 1 time per week or more. Mothers who consumed dried beans were further asked, “*Each time you ate beans, how much did you usually eat?”* The initial answer choices of portion size included less than ½ cup, ½ cup to 1 cup, and more than 1 cup. For our analysis, we calculated the total weekly amount of dried bean consumption as the product of frequency and portion size (i.e., amount [cups/week] = frequency [times per week] × portion size [cups per time]). To facilitate calculation, the original frequency options of dried bean consumption were numerically converted into values with the same unit (times/week), i.e., 0 times/week for never, 0.230137 times/week for 1 time per month, 0.575343 times/week for 2–3 times per month (mid-point), 1 time/week for 1 time per week, 2 times/week for 2 times per week, 3.5 times/week for 3–4 times per week (mid-point), 5.5 times/week for 5–6 times per week (mid-point), 7 times/week for 1 time per day, and 14 times/week for 2 or more times per day (the lower boundary for this high-frequency category). Portion size options were altered to 0 cups/time for no consumption, 0.25 cups/time for less than ½ cup (mid-point), 0.75 cups/time for ½ to 1 cup (mid-point), and 1 cup/time for more than 1 cup (the lower boundary for this large-portion category).

For chili consumption, mothers were asked, “*How often did you eat chili?”*, with initial answer choices being never, 1 time per month, 2–3 times per month, 1 time per week, 2 times per week, 3–4 times per week, 5–6 times per week, 1 time per day, and 2 or more times per day. Answer choices were regrouped to never, 1 time per month, and 2–3 times per month or more. Mothers who consumed chili were further asked, “*Each time you ate chili, how much did you usually eat?”* Initial answer choices included less than ½ cup, ½ to 1¾ cups, and more than 1¾ cups. The total weekly amount of chili consumption was calculated as the product of frequency and portion size (i.e., amount [cups/week] = frequency [times per week] × portion size [cups per time]). The frequency of chili consumption was numerically converted into 0 times/week for never, 0.230137 times/week for 1 time per month, 0.575343 times/week for 2–3 times per month (mid-point), 1 time/week for 1 time per week, 2 times/week for 2 times per week, 3.5 times/week for 3–4 times per week (mid-point), 5.5 times/week for 5–6 times per week (mid-point), 7 times/week for 1 time per day, and 14 times/week for 2 or more times per day (the lower boundary for this high-frequency category). Portion size options were converted into 0 cups for no consumption, 0.25 for less than ½ cup (mid-point), 1.125 for ½ to 1¾ cups (mid-point), and 1.75 for more than 1¾ cups (the lower boundary for this large-portion category).

For soup consumption, mothers were asked, “*How often did you eat soups?”* Initial answer choices were never, 1 time per month, 2–3 times per month, 1 time per week, 2 times per week, 3–4 times per week, 5–6 times per week, 1 time per day, and 2 or more times per day. Mothers who consumed soup were further asked, “*Each time you ate soup, how much did you usually eat?”*, with answer choices being less than 1 cup, 1 to 2 cups, and more than 2 cups. The options were altered to 0 cups for no consumption, 0.5 for less than 1 cup (mid-point), 1.5 for 1 to 2 cups (mid-point), and 2 for more than 2 cups (the lower boundary for this large-portion category). Mothers were then asked, “*How often were the soups you ate bean soups?”* Answer choices were almost never or never (0%), about ¼ of the time (25%), about ½ of the time (50%), about ¾ of the time (75%), and almost always or always (100%). Accordingly, we calculated the frequency of bean soup consumption as the frequency of total soup consumption × the proportion of bean soups consumed over the total soups. Then, the weekly amount of bean soup consumption was calculated as the frequency of total soup consumption × the portion size of total soup consumption × the proportion of bean soups consumed over the total soups.

To reduce potential bias of self-reported dietary intake data, some participants were excluded from the analytic sample due to their extreme calorie report (Figure 1), according to data cleaning and validation approaches recommended by Willett [40]. Specifically, the National Cancer Institute (NCI) staff examined the distributions of estimated energy intake to identify extreme values that were physiologically implausible and substantially discrepant from a normal distribution. Accordingly, they excluded 48 pregnant mothers at the bottom 1% (<671.12 kilocalories) or top 2% (>6264.61 kilocalories) of total energy intake. After these exclusions, the natural logarithms of total energy intake, protein, fat, and carbohydrates were normally distributed.

### 2.3. Outcome Measures

Pregnant women in their third trimester were mailed a prenatal questionnaire. Specifically, mothers were asked to fill out either “yes”, “no”, or “don’t know” in answer to the question, “*Have you had gestational diabetes with this pregnancy?”.* A “yes” response to this question was defined as the existence of GDM, a “no” response was defined as the absence of GDM, and a “don’t know” response was coded as missing data. Note that IFPS II lacked clinical diagnostic data on GDM. Previous research suggests that the questionnaire-based reporting of GDM seemed to have acceptable validity [41,42]. For example, in a previous study, 78 of the 82 women with GDM diagnosis by an oral glucose tolerance test correctly reported having GDM through a questionnaire survey, giving an overall sensitivity of 95.1%; and all 83 women without GDM diagnosis correctly reported not having GDM, indicating 100% specificity [41].

### 2.4. Correlates of Exposures and Outcomes

Based on the previous literature [18,43,44,45,46,47,48], we considered several important socio-demographic and pregnancy-related characteristics as potential confounders in the association between maternal bean consumption and the risk of GDM. These characteristics included household poverty level, size, and annual income, and maternal age, race/ethnicity, highest education level, employment status, enrollment of WIC, geographical region, Healthy Eating Index (HEI), and cigarette smoking status during pregnancy. By using the recommendations of the USDA Dietary Guidelines for Americans, we calculated the maternal HEI-2005 during pregnancy. HEI-2005 consists of 9 adequacy components: total fruit (5 points), whole fruit (5 points), total vegetables (5 points), dark green and orange vegetables and legumes (5 points), total grains (5 points), whole grains (5 points), milk (10 points), meat and beans (10 points), and oils (10 points). It also consists of 3 moderation components: saturated fat (10 points), sodium (10 points), and calories from solid fats, alcoholic beverages (i.e., beer, wine, and distilled spirits), and added sugars (20 points) [49]. A total score 100 is used for HEI-2005, with a higher score representing a higher-quality diet [49]. Including the HEI as a correlate could help control the confounding effect of overall dietary quality and other food intake in nutritional epidemiological research [50].

### 2.5. Statistical Analysis

A descriptive analysis was used to summarize participants’ characteristics: frequencies and percentages for categorical variables (e.g., race) and means and standard deviations (SDs) for continuous variables (e.g., age) (the table in Section 3.1). We used analysis of variance (ANOVA) and Tukey’s honestly significant difference test to determine if the mean amount of bean consumption during pregnancy (cups/week) differed by each socio-demographic and pregnancy-related characteristic (the table in Section 3.2). Chi-square tests were used to compare the risk of GDM (outcome) by categorical correlates (the table in Section 3.3). For continuous correlates, we used *t*-tests to compare the means of women with GDM and those without. We used multivariable logistic regression models to examine associations between maternal bean consumption (exposure) and risk of GDM (outcome) (the table in Section 3.4). We chose logistic regression to fit the binary outcome of GDM, given its advantages of simultaneous adjustment for confounders measured on different scales, clinically interpretable estimates, and validity in different study designs with few underlying assumptions [51,52]. Crude models explored the association between each bean food consumed (dried bean, chili, or bean soup) and GDM status (GDM vs. no GDM). Adjusted models examined this association after adjusting for socio-demographic and pregnancy-related confounders. For both crude and adjusted models, the odds ratios (ORs) of GDM and their 95% confidence interval (CI) were calculated for the frequency of dried bean, chili, or bean soup consumption, with the reference group being “never consumed”. In addition, we calculated the ORs and their 95% CI corresponding to 1 cup/week increment of the amount (continuous variables) of dried bean, chili, or bean soup consumption. Given the substantial disparities in the risk of GDM and prenatal care by socio-demographics (e.g., race/ethnicity, age, education attainment, and WIC recipient status) [53,54,55,56,57,58], we conducted supplemental analyses to test the potential interactions between these socio-demographic characteristics and bean consumption. All data analyses were performed by using SAS 9.4 (SAS Institute, Cary, NC, USA). We defined statistical significance as a two-sided *p*-value < 0.05. 

## 3. Results

### 3.1. Sample Characteristics

The socio-demographic and pregnancy-related characteristics of the analytical sample (N = 1397) are presented in Table 1. The average age of mothers was 28.8 (SD, 5.6) years. Of all mothers, 83.8% were non-Hispanic White, 4.8% were non-Hispanic Black, 6.7% were Hispanic, and 4.7% were of other races. Most mothers attended college (78.8%), 64.3% were employed, 74.4% had a household size of three or more people, 32.5% had a household income of ≥U.S. dollar (USD) 60,000, 41.1% were WIC recipients, and 89.3% did not smoke cigarettes during pregnancy. The distribution of age, race/ethnicity, and region varied by the level of dried bean consumption (Table A2). The distribution of race/ethnicity, education, and region varied by the level of chili consumption (Table A3). The distribution of race/ethnicity, college education, and employment status varied by the level of bean soup consumption (Table A4).

### 3.2. Distribution and Correlates of Bean Consumption

Table 2 provides the amount of bean consumption during pregnancy based on the socio-demographic and pregnancy-related characteristics. Pregnant women consumed an average of 0.31 cups/week of dried beans, 0.16 cups/week of chili, and 0.10 cups/week of bean soup. The average total amount (0.57 cups/week) of dried beans, chili, and bean soup combined was lower than (about 38%) the recommended 1.5 cups/week of dietary pulses in the Dietary Guidelines for Americans 2020–2025 [16]. Maternal dried bean consumption significantly varied by race (*p*-value < 0.001) and geographic region (*p*-value < 0.001). On average, Hispanic mothers reported more dried bean consumption (mean, 0.65 cups/week) than other racial/ethnic groups (0.25–0.33 cups/week). Mothers in the East South Central region had the highest consumption of dried beans (0.44 cups/week). There were no significant differences in dried bean consumption by other characteristics, including education level, employment status, household size, household income, WIC recipient status, and smoking status during pregnancy.

Chili consumption during pregnancy varied significantly by maternal race (*p*-value < 0.001), education level (*p*-value = 0.011), household size (*p*-value = 0.005), household income (*p*-value = 0.032), WIC recipient status (*p*-value = 0.016), and region (*p*-value = 0.034). Chili consumption was the highest among non-Hispanic Black mothers, averaging 0.33 cups/week. Mothers who had not attended college consumed more chili (0.18 cups/week) than mothers who had attended college (0.14 cups/week). The lowest amount of chili consumption was reported by mothers living in a household with three people (0.11 cups/week) and those who had an annual household income of ≥USD 60,000 (0.13 cups/week). Mothers who were WIC recipients consumed more chili (0.18 vs. 0.14 cups/week) than non-WIC recipients. Mothers in the East South Central region reported the highest consumption of chili (0.26 cups/week), while mothers in the Middle Atlantic or New England region reported the lowest consumption of chili (0.10 cups/week). Bean soup consumption did not vary significantly by any of the socio-demographic and pregnancy-related characteristics of interest.

### 3.3. Correlates of GDM

The risk of GDM by socio-demographic and pregnancy-related characteristics is provided in Table 3. Mothers with GDM were, on average, 2.6 years older than their counterparts without GDM (*p*-value < 0.001). Cigarette smoking during pregnancy was also a significant risk factor for GDM (11.4% among smokers vs. 6.3% among non-smokers; *p*-value = 0.019).

### 3.4. Association Between Bean Consumption and GDM

Table 4 shows the association between bean consumption during pregnancy and the risk of GDM. Mothers who consumed chili one time per month had a lower risk of GDM (3.5% vs. 7.4%), compared with never consumers. The crude OR was 0.46 (95% CI, 0.24–0.87; *p*-value = 0.018). After adjustment for socio-demographic and pregnancy-related confounders, this association remained significant, with the confounder-adjusted OR being 0.37 (95% CI, 0.17–0.79; *p*-value = 0.011). However, mothers who consumed chili two–three times per month or more did not have significantly different risk of GDM from never consumers (10.1% vs. 7.4%; crude OR, 1.40 [95% CI, 0.81–2.41]; *p*-value = 0.233). Adjustment for confounders had little impact on the association, i.e., the confounder-adjusted OR was 1.41 (95% CI, 0.77–2.57; *p*-value = 0.266). When the amount of chili consumption was used as a continuous variable, its association with the risk of GDM was not statistically significant (confounder-adjusted OR, 1.42 [95% CI, 0.84–2.40]; *p*-value = 0.196). To assess the robustness of the observed associations between moderate chili consumption (one time per month vs. never) and the risk of GDM, we conducted a sensitivity analysis by assuming different levels of report bias of GDM. The corrected crude OR was 0.47 (95% CI, 0.23–0.96; *p*-value = 0.034) when assuming a 20% over-reporting of GDM, 0.47 (95% CI, 0.24–0.92; *p*-value = 0.025) when assuming a 10% over-reporting of GDM, 0.45 (95% CI, 0.24–0.84; *p*-value = 0.01) when assuming a 10% under-reporting of GDM, and 0.45 (95% CI, 0.25–0.82; *p*-value = 0.007) when assuming a 20% under-reporting of GDM, respectively.

Dried bean and bean soup consumption was not associated with the risk of GDM. This was the case for both categorical and continuous variables of consumption. For example, mothers who consumed dried beans one time per week or more had a similar risk of GDM (6.6% vs. 7.0%; confounder-adjusted OR, 0.82 [95% CI, 0.41–1.62]; *p*-value = 0.569) to mothers who never consumed dried beans. Mothers who consumed bean soup two–three times per month or more had a similar risk of GDM (4.9% vs. 6.6%; confounder-adjusted OR, 0.40 [95% CI, 0.05–3.08]; *p*-value = 0.382) to mothers who never consumed bean soup.

In the supplemental analyses to test the potential interactions between socio-demographic characteristics (e.g., race/ethnicity, age, education attainment, and WIC recipient status) and bean consumption, we found that these interaction terms in the regression models were either statistically non-significant or clinically non-meaningful, and some were not even estimable due to the small sample size of the sub-categories.

In addition, we ran a post hoc statistical power analysis to assess the adequacy of our sample size for examining the associations between bean consumption and GDM. For chili consumption, the statistical power was 70.7% for the comparison between one time per month vs. never consumed, 21.2% for the comparison between two–three times per month or more vs. never consumed, and 18.0% for the continuous variable in cups/week. The corresponding power values were 4.3% (one time per month), 4.6% (two–three times per month), 4.3% (one time per week or more), and 5.1% (continuous), respectively, for dried bean consumption and 23.5% (one time per month), 0.8% (two–three times per month or more), and 5.7% (continuous), respectively, for bean soup consumption.

## 4. Discussion

By using data from U.S. pregnant women in a longitudinal pre-birth cohort study, we examined the associations of bean consumption with the risk of GDM. We found that bean consumption was low among pregnant women, with substantial socio-demographic variation in consumption. Maternal consumption of chili one time per month was associated with a low risk of GDM. However, there was no association between dried bean or bean soup consumption and the risk of GDM. These novel findings may help identify disparities in bean consumption during pregnancy and understand how bean consumption may contribute to the risk of GDM.

In this study, we found that bean consumption among pregnant women was low, like in the general U.S. population [44,59]. Specifically, their average total amount (0.57 cups/week) of dried beans, chili, and bean soup combined was lower than the recommended 1.5 cups/week of dietary pulses as recommended in the Dietary Guidelines for Americans 2020–2025 [16]. Data from the National Health and Nutrition Examination Survey (NHANES) 1999–2000 [60] indicate that adults consume ≤ 1/3 of the recommended amount of legumes (which include dried beans). Furthermore, from 1999 to 2002, only about 7.9% of the U.S. population consumed beans daily [61]. The overall low consumption of beans in the U.S. suggests that beans are not considered a major component of the American diet [59]. One possible explanation is a lack of access to individuals’ favored bean type, form (dried and canned), and packaging in certain U.S. regions [45]. This is unfortunate, as increased bean intake could help improve the overall dietary quality in pregnancy and can help fill some of the current nutrition gaps among pregnant women, such as fiber and micronutrient intake.

We also observed a considerable variation in the consumption of dried beans by race/ethnicity. Mothers of Hispanic origin had the highest dried bean consumption across all racial/ethnic groups. According to Lucier et al. [43], Hispanics consumed the highest amount of cooked dried beans and were also high consumers of many other types of pulses. Furthermore, although Hispanics make up only 11.4% of the U.S. population, they account for 33% of people who regularly consume cooked dried beans [43]. The higher dried bean consumption among Hispanics is likely due to the common use of beans in many Latin American dishes, as well as their widespread availability and low prices in local markets [59]. Moreover, dried beans can easily be stored for long periods of time [18]. All these factors together make dried beans an easy and affordable option for Hispanic mothers to incorporate a nutrient-rich ingredient into various meals. Within the U.S., dried bean consumption is the highest in the southern and western states [43]. This is consistent with our observation that mothers who lived in the East South Central and West South Central regions consumed the highest amount of dried beans. The higher dried bean consumption within these geographic areas may be due to the presence of a large Hispanic population [43].

In our study population, dried bean consumption was the highest among mothers with postgraduate-level education. A similar association was also observed in a previous study in the general U.S. population [62]. This could be due to a lack of knowledge of the health benefits of bean consumption in people with lower education. Promisingly, research has shown that intervention to improve the understanding of the health benefits of beans among low-income women could increase bean consumption and improve nutritional outcomes [63].

In contrast, chili consumption was the highest among mothers with the lowest level of education (1–8 years of grade school) in our study. Moreover, mothers with the lowest income level reported having the highest chili consumption. Although the reasons for these differences are unknown, we proposed several potential explanations. First, chili is an affordable and easily accessible food item. Chili is highly affordable (e.g., approximately 0.25 USD cents/serving) [45] and widely available at various stores. Chili can be made in little time with other affordable ingredients. Chili can also be easily incorporated into other meals as a side dish to enhance meal flavor and be made in large portions for future consumption. These advantages make chili appealing to mothers with low incomes, as they may have very limited money, time, and energy to prepare foods [64]. In addition, the canned option of chili has a long shelf life and can serve as a stable food source for low-income individuals who may experience food insecurity [45].

We observed a significant association between moderate chili consumption and a lower risk of GDM in U.S. mothers with diverse socio-demographics. This finding was similar to a previous study conducted in a developing country (Iran) reporting that individuals who ate ≥ 3.3 servings of legumes per week before pregnancy or during early pregnancy had lower odds of GDM, compared with individuals who ate ≤ 1.5 servings per week [37]. In another study from a developed country (Czechia), the frequency of legume consumption during early pregnancy was found to be inversely correlated with the level of fasting plasma glucose during the second trimester of pregnancy [65]. Additionally, researchers reported a lower risk of GDM associated with certain dietary patterns that contain beans and other legumes. For example, U.S. Nurses’ Health Study II showed that a stronger adherence to the Mediterranean diet (the fourth quartile vs. the first quartile), which encourages high consumption of legumes, was associated with a 24% lower risk of GDM [35]. Two other studies in Chinese women also observed an association between a vegetable dietary pattern (involving beans or bean products) and a low risk of GDM [33,66]. In a similar study in middle-aged Chinese women (non-pregnant), Villegas et al. identified an association between a diet containing high consumption of legumes (e.g., soybean, peanuts, and other legumes) and a low risk of type 2 diabetes mellitus [31].

Our observed negative association between chili consumption and GDM risk may be attributed to several factors, from both biological (e.g., fiber [67], resistant starch [23], phytochemicals [28], and capsaicin [68]) and non-biological (e.g., cultural) perspectives. First, previous work has shown that pulse or legume consumption improved markers of glycemic control, including glycated hemoglobin A1c (HbA1c), fasting blood glucose, and fasting insulin [20,21,22,23,69]. This could be explained by pulses’ high dietary fiber content, ranging from 11.5% to 33.2% of dry matter [70]. Dietary fiber has been shown to decrease postprandial serum glucose by interfering with intestinal glucose absorption by increasing the viscosity of the intestinal contents [67] or by inhibiting α-amylase activity and delaying starch breakdown [71]. Additionally, Miehle et al. reported that soluble dietary fibers with strong hydration properties also decreased glucose diffusion [72]. Further, the resistant starch content of pulses resists digestion in the small intestine and enters the large intestine as a fermentable energy source for resident bacteria [27,73]. Resistant starch has been found to increase serum short-chain fatty acid (SCFA) concentrations (i.e., acetate, butyrate, and propionate) in many animal studies, and a moderate amount has been associated with improved glucose responses [27,74,75,76,77]. Thompson et al. assumed that a significantly lowered glucose response after the consumption of black and pinto beans was due to their higher levels of indigestible starch when compared with kidney beans [23]. They reasoned that this would slow down digestion and therefore glucose absorption [23]. Secondly, beans contain phytochemicals, such as phenolic compounds and phytates, that could potentially improve glycemic control. In a previous rat study, polyphenols were shown to interfere with some glucose transporters, thus interfering with glucose absorption [29]. Phenolic compounds have also been correlated with the inhibition of α-amylase and α-glucosidase, slowing glucose digestion [78]. Darker-colored beans (e.g., black, red, pink, and brown), a common ingredient in chili, have been found to contain greater amounts of total phenolic compounds [28]. Another phytochemical in pulses, phytate, has been found to lower blood glucose levels in animal studies [79]. A third possible explanation may pertain to the glucose-lowering effect of capsaicin, a plant-based bioactive compound that is often used in chili as a component of chili powder and paprika [80]. Other chili recipe variations may use cayenne pepper or jalapeno peppers, both of which contain capsaicin. Previous work has reported that capsaicin-containing chili improved postprandial hyperglycemia in women with GDM [68]. Similarly, another study found that capsaicin from the *Capsicum frutescens* plant was associated with a decrease in plasma glucose levels in healthy volunteers [81]. Lastly, we cannot rule out the possibility that the estimated association between chili consumption and GDM risk in this observational cohort study might be partially due to overall dietary patterns or cultural factors related to chili consumption.

Interestingly, we found that chili appeared only protective against GDM when consumed one time per month. However, a higher frequency (≥two times per month) of chili consumption was not significantly associated with GDM. This response may be related to other ingredients in chili, such as red meat. Red meat is a common ingredient in chili in the form of ground beef [80,82]. Previous work suggests that red meat is associated with an increased risk of GDM [83,84,85]. Specifically, the heme iron, saturated fatty acid, and cholesterol components of red meat have been shown to promote insulin resistance and lead to either a higher risk of GDM or type 2 diabetes [83,84,86,87]. Therefore, the potentially protective effects of chili due to bean or capsaicin components may be blunted with a higher frequency of intake, as red meat intake is also increased.

Unexpectedly, we did not observe a statistically significant association between consuming dried beans or bean soup and GDM risk. Potential differences in the risk of GDM between chili versus dried bean and bean soup can be attributed to differences in their recipe components. As mentioned previously, the capsaicin component of chili may confer some of its benefits to GDM prevention [68], whereas many dried bean or bean soup recipes do not contain capsaicin [88]. Further, different types of beans may influence GDM risk because of their varying amounts of phytochemicals, which have been shown to affect glucose metabolism [28,29,78]. USDA recipes call for pinto beans for chili and great northern beans for bean soup, whereas dried beans can be any type of beans [80,89]. Additionally, the non-significant associations between dried beans and bean soup might be due to dietary variations in their preparation methods, including fat and water contents, and other ingredients, such as vegetables and meats. Lastly, the differences in results across the three bean foods could be related to our methodological limitations, especially random errors due to overall low bean consumption and recall bias of bean consumption.

### Limitations and Strengths

Our study had several important limitations. Firstly, the use of Diet History Questionnaire could lead to the inaccurate self-reporting of bean consumption over the previous month (the only time point during pregnancy). Similar bias could occur in the reported diagnosis of GDM. We suspect these misclassifications in bean consumption (exposure) or GDM (outcome) were likely to be non-differential (independent from each other) and thus bias the estimated associations toward null [90]. Secondly, the analytic sample had a higher percentage of non-Hispanic White mothers who were more educated and had a higher household income than the excluded sample. This selection bias could affect the external validity (generalizability) of our results, especially the prevalence statistics. Thirdly, detailed information was lacking on the types of beans (e.g., black, pinto, and kidney) and other ingredients (e.g., meat) used in the chili and bean soup. Fourthly, caution is needed to interpret the non-causal results from our observational study without intervention. Fourthly, we did not have direct biochemical evidence to support the negative association between moderate chili consumption and the risk of GDM, which warrants further investigation in future research. In particular, it is necessary to separate meat-containing and vegetarian chili. Similarly, future studies in this field must carefully measure dried bean and bean soup preparation methods, which can provide nuances on their nutrient and health effects. Fifthly, the lack of information on physical activity in IFPS II did not allow us to adjust for this important behavioral factor for GDM [91]. Lastly, the estimated associations in this observational cohort study should be interpreted as suggestive rather than causal, and a randomized controlled trial is needed to confirm our findings.

However, our study also had multiple strengths. Firstly, the relatively large sample size helped us obtain reliable estimates of bean consumption and risk of GDM. Secondly, we separated the consumption of different bean foods (i.e., dried beans, chili, and bean soup), especially given that they might have differential health effects. Thirdly, we adjusted for key socio-demographic and pregnancy-related confounders in the regression models.

## 5. Conclusions

In summary, our research indicates overall low bean consumption during pregnancy among U.S. women and substantial socio-demographic disparities in bean consumption. A moderate frequency of chili consumption during pregnancy was associated with a low risk of GDM. Our findings should be interpreted cautiously, especially given the non-causal nature of observational research, recall bias, and selection bias. Replication is needed in larger cohorts with more diverse populations, detailed measures of bean consumption repeatedly at several time points during pregnancy (e.g., early, middle, and late), gold standards of GDM diagnosis, and experimental design. Research in this field has the potential to inform dietary approaches to addressing GDM and related morbidities, an increasing maternal and child health concern in the U.S. and globally.

## Figures and Tables

**Table 1:** Socio-demographic and pregnancy-related characteristics of participants in the analytic sample.

	Analytic Sample (N = 1397)	
Characteristics *	n (%[95% CI])	Mean ± SD
**Age, years**		28.8 ± 5.6
**% of federal poverty level**		257.5 ± 189.0
**Race/ethnicity**		
Non-Hispanic White	1161 (83.8 [81.8–85.7])	
Non-Hispanic Black	67 (4.8 [3.7–6.0])	
Hispanic	93 (6.7 [5.4–8.0])	
Non-Hispanic Asian/Pacific Islander/other	65 (4.7 [3.6–5.8])	
**Highest education level**		
1–8 years of grade school	4 (0.3 [0.0–0.6])	
High school	271 (20.9 [18.7–23.1])	
1–3 years of college	519 (40.0 [37.4–42.7])	
College graduate	383 (29.5 [27.1–32.0])	
Postgraduate	120 (9.3 [7.7–10.8])	
**College education**		
Did not attend college	275 (21.2 [19.0–23.4])	
Attended college	1022 (78.8 [76.6–81.0])	
**Mothers’ employment status**		
Unemployed	496 (35.7 [33.1–38.2])	
Employed	895 (64.3 [61.8–66.9])	
**Household size**		
1–2 people	358 (25.6 [23.3–27.9])	
3 people	527 (37.7 [35.2–40.3])	
4 people	282 (20.2 [18.1–22.3])	
5+ people	230 (16.5 [14.5–18.4])	
**Annual household income level**		
<USD 25,000	312 (22.3 [20.2–24.5])	
USD 25,000–<USD 40,000	314 (22.5 [20.3–24.7])	
USD 40,000–<USD 60,000	317 (22.7 [20.5–24.9])	
≥USD 60,000	454 (32.5 [30.0–35.0])	
**WIC recipient status**		
Non-recipient	823 (58.9 [56.3–61.5])	
Recipient	574 (41.1 [38.5–43.7])	
**Region**		
New England	68 (4.9 [3.7–6.0])	
Middle Atlantic	165 (11.8 [10.1–13.5])	
East North Central	281 (20.1 [18.0–22.2])	
West North Central	136 (9.7 [8.2–11.3])	
South Atlantic	230 (16.5 [14.5–18.4])	
East South Central	79 (5.7 [4.4–6.9])	
West South Central	151 (10.8 [9.2–12.4])	
Mountain	136 (9.7 [8.2–11.3])	
Pacific	151 (10.8 [9.2–12.4])	
**Smoking during pregnancy**		
No	1240 (89.3 [87.7–90.9])	
Yes	149 (10.7 [9.1–12.4])	

SD, standard deviation. * For some characteristics, the sum of categories is less than the total due to missing data.

**Table 2:** Maternal bean consumption during pregnancy by socio-demographic and pregnancy-related characteristics.

	Dried Bean			Chili			Bean Soup		
Characteristics	Mean ± SD (Cups/Week)	Overall *p*-Value *	Pairwise Comparisons **	Mean ± SD (Cups/Week)	Overall *p*-Value *	Pairwise Comparisons **	Mean ± SD (Cups/Week)	Overall *p*-Value *	Pairwise Comparisons **
**Overall**	0.31 ± 0.57			0.16 ± 0.35			0.10 ± 0.36		
**Race/ethnicity**		**<0.001**			**<0.001**			0.832	
Non-Hispanic White	0.28 ± 0.49		a	0.14 ± 0.28		a	0.10 ± 0.34		
Non-Hispanic Black	0.25 ± 0.38		a	0.33 ± 0.90		b	0.09 ± 0.41		
Hispanic	0.65 ± 1.06		b	0.15 ± 0.35		a	0.13 ± 0.36		
Non-Hispanic Asian/Pacific Islander/other	0.33 ± 0.56		a	0.18 ± 0.31		a,b	0.11 ± 0.36		
**Education level**		0.219			**0.011**			0.214	
1–8 years of grade school	0.12 ± 0.21			0.57 ± 0.41		a	0.05 ± 0.11		
High school	0.28 ± 0.61			0.18 ± 0.37		a,b	0.07 ± 0.25		
1–3 years of college	0.28 ± 0.46			0.15 ± 0.37		a,b	0.08 ± 0.29		
College graduate	0.30 ± 0.52			0.11 ± 0.23		b	0.12 ± 0.45		
Postgraduate	0.41 ± 0.79			0.13 ± 0.33		a,b	0.12 ± 0.30		
**College education**		0.517			**0.045**			0.176	
Did not attend college	0.28 ± 0.60			0.18 ± 0.37		a	0.07 ± 0.25		
Attended college	0.30 ± 0.53			0.14 ± 0.32		b	0.10 ± 0.36		
**Mothers’ employment status**		0.382			0.332			0.615	
Unemployed	0.33 ± 0.62			0.17 ± 0.36			0.09 ± 0.29		
Employed	0.30 ± 0.54			0.15 ± 0.34			0.10 ± 0.38		
**Household size**		0.179			**0.005**			0.127	
1–2 people	0.32 ± 0.54			0.17 ± 0.43		a, b	0.13 ± 0.49		
3 people	0.28 ± 0.56			0.11 ± 0.23		a	0.09 ± 0.29		
4 people	0.29 ± 0.49			0.19 ± 0.40		b	0.07 ± 0.27		
5+ people	0.38 ± 0.71			0.19 ± 0.34		b	0.13 ± 0.35		
**Annual household income level**		0.278			**0.032**			0.841	
<USD 25,000	0.26 ± 0.52			0.20 ± 0.51		a	0.11 ± 0.33		
USD 25,000–<USD 40,000	0.35 ± 0.63			0.14 ± 0.23		a, b	0.11 ± 0.44		
USD 40,000–<USD 60,000	0.32 ± 0.59			0.15 ± 0.28		a, b	0.10 ± 0.31		
≥USD 60,000	0.31 ± 0.54			0.13 ± 0.32		b	0.09 ± 0.34		
**WIC recipient status**		0.567			**0.016**			0.765	
Non-recipient	0.32 ± 0.57			0.14 ± 0.29		a	0.10 ± 0.36		
Recipient	0.30 ± 0.58			0.18 ± 0.41		b	0.10 ± 0.35		
**Region**		**<0.001**			**0.034**			0.597	
New England	0.26 ± 0.64		a, b, c	0.10 ± 0.24		a, b	0.13 ± 0.51		
Middle Atlantic	0.31 ± 0.71		a, b, c	0.10 ± 0.26		a	0.14 ± 0.48		
East North Central	0.22 ± 0.41		a, b	0.15 ± 0.26		a, b	0.07 ± 0.22		
West North Central	0.15 ± 0.29		a	0.14 ± 0.31		a, b	0.07 ± 0.32		
South Atlantic	0.36 ± 0.61		b, c	0.15 ± 0.52		a, b	0.10 ± 0.39		
East South Central	0.44 ± 0.56		b, c	0.26 ± 0.36		b	0.11 ± 0.33		
West South Central	0.40 ± 0.63		c	0.16 ± 0.27		a, b	0.10 ± 0.37		
Mountain	0.32 ± 0.57		a, b, c	0.19 ± 0.36		a, b	0.11 ± 0.31		
Pacific	0.38 ± 0.65		b, c	0.19 ± 0.35		a, b	0.13 ± 0.35		
**Smoking during pregnancy**		0.135			0.616			0.350	
No	0.32 ± 0.58			0.15 ± 0.35			0.10 ± 0.37		
Yes	0.24 ± 0.48			0.17 ± 0.35			0.08 ± 0.28		

SD, standard deviation. * Analysis of variance. Significant *p*-values (<0.05) are in bold. ** Tukey’s honestly significant difference test. If two groups do not share any letters, they have significantly different mean values of bean consumption.

**Table 3:** Risk of gestational diabetes by socio-demographic and pregnancy-related characteristics.

		Risk of Gestational Diabetes		
Characteristics *	Sample Size, N	n (% [95% CI])	Mean Difference ± SE **	*p*-Value ***
**Age, years**	1391		2.6 ± 0.6	**<0.001**
**% of federal poverty level**	1397		38.5 ± 20.0	0.144
**Race/ethnicity**				0.862
Non-Hispanic White	1161	82 (7.1 [5.6–8.5])		
Non-Hispanic Black	67	3 (4.5 [0.0–9.4])		
Hispanic	93	6 (6.5 [1.5–11.4])		
Non-Hispanic Asian/Pacific Islander/other	65	5 (7.7 [1.2–14.2])		
**Highest education level**				0.461
1–8 years of grade school	4	0 (0.0 [0.0–0.0])		
High school	271	19 (7.0 [4.0–10.1])		
1–3 years of college	519	32 (6.2 [4.1–8.2])		
College graduate	383	26 (6.8 [4.3–9.3])		
Postgraduate	120	13 (10.8 [5.3–16.4])		
**College education**				0.982
Did not attend college	275	19 (6.9 [3.9–9.9])		
Attended college	1022	71 (7.0 [5.4–8.5])		
**Mothers’ employment status**				0.696
Unemployed	496	36 (7.3 [5.0–9.5])		
Employed	895	60 (6.7 [5.1–8.3])		
**Household size**				0.408
1–2 people	358	26 (7.3 [4.6–10.0])		
3 people	527	39 (7.4 [5.2–9.6])		
4 people	282	13 (4.6 [2.2–7.1])		
5+ people	230	18 (7.8 [4.4–11.3])		
**Annual household income level**				0.326
<25,000	312	23 (7.4 [4.5–10.3])		
25,000–<40,000	314	15 (4.8 [2.4–7.1])		
40,000–<60,000	317	21 (6.6 [3.9–9.4])		
≥60,000	454	37 (8.2 [5.6–10.7])		
**WIC recipient status**				0.599
Non-recipient	823	59 (7.2 [5.4–8.9])		
Recipient	574	37 (6.5 [4.4–8.5])		
**Region**				0.557
New England	68	4 (5.9 [0.3–11.5])		
Middle Atlantic	165	13 (7.9 [3.8–12.0])		
East North Central	281	18 (6.4 [3.5–9.3])		
West North Central	136	10 (7.4 [3.0–11.7])		
South Atlantic	230	15 (6.5 [3.3–9.7])		
East South Central	79	3 (3.8 [0.0–8.0])		
West South Central	151	15 (9.9 [5.2–14.7])		
Mountain	136	5 (3.7 [0.5–6.8])		
Pacific	151	13 (8.6 [4.1–13.1])		
**Smoking during pregnancy**				**0.019**
No	1240	78 (6.3 [4.9–7.6])		
Yes	149	17 (11.4 [6.3–16.5])		

SE: Standard error. * For some characteristics, the sum of categories is less than the total due to missing data. ** Mean difference = mean characteristic of mothers with gestational diabetes − mean characteristic of mothers without gestational diabetes. *** *p*-Values from Chi-square tests for categorical correlates and *t*-test for continuous correlates. Significant *p*-values (<0.05) are in bold.

**Table 4:** Associations between maternal bean consumption during pregnancy and the risk of gestational diabetes.

Maternal Bean Consumption During Pregnancy		Risk of Gestational Diabetes				
	Sample Size, N	n (% [95% CI])	Crude OR (95% CI)	Crude OR *p*-Value	Adjusted OR (95% CI) *	Adjusted OR *p*-Value
**Frequency of dried bean consumption**						
Never	633	44 (7.0 [5.0–8.9])	Reference		Reference	
1 time per month	214	14 (6.5 [3.2–9.9])	0.94 (0.50–1.75)	0.838	0.93 (0.48–1.79)	0.834
2–3 times per month	320	23 (7.2 [4.4–10.0])	1.04 (0.61–1.75)	0.893	1.01 (0.58–1.78)	0.969
1 time per week or more	229	15 (6.6 [3.4–9.8])	0.94 (0.51–1.72)	0.837	0.82 (0.41–1.62)	0.569
**Amount of dried bean consumption, 1 cup/week increment**	1394		0.98 (0.68–1.42)	0.930	0.82 (0.51–1.34)	0.430
**Frequency of chili consumption**						
Never	903	67 (7.4 [5.7–9.1])	Reference		Reference	
1 time per month	312	11 (3.5 [1.5–5.6])	**0.46 (0.24–0.87)**	**0.018**	**0.37 (0.17–0.79)**	**0.011**
2–3 times per month or more	179	18 (10.1 [5.7–14.5])	1.40 (0.81–2.41)	0.233	1.41 (0.77–2.57)	0.266
**Amount of chili consumption, 1 cup/week increment**	1393		1.36 (0.87–2.12)	0.176	1.42 (0.84–2.40)	0.196
**Frequency of bean soup consumption**						
Never	1140	75 (6.6 [5.1–8.0])	Reference		Reference	
1 time per month	211	19 (9.0 [5.1–12.9])	1.41 (0.83–2.38)	0.205	1.30 (0.72–2.33)	0.380
2–3 times per month or more	41	2 (4.9 [0.0–11.5])	0.73 (0.17–3.07)	0.666	0.40 (0.05–3.08)	0.382
**Amount of bean soup consumption, 1 cup/week increment**	1392		0.93 (0.50–1.73)	0.817	0.76 (0.33–1.72)	0.505

OR, odds ratio; CI, confidence interval. * Adjusted for household poverty level, size, and annual income; maternal age, race/ethnicity, education, and employment status, WIC enrollment, region, Healthy Eating Index, and cigarette smoking status during pregnancy. Significant *p*-values (<0.05) are in bold.

## Data Availability

The data described in the manuscript, code book, and analytic code will not be made available because the data were provided by the CDC and cannot be shared without permission. Researchers who are interested in using the IFPS II data can contact the CDC directly (ifps@cdc.gov).

## Abbreviations

The following abbreviations are used in this manuscript:

GDM	gestational diabetes mellitus
USDA	United States Department of Agriculture
iAUC	incremental area under the glycemic response curve
DASH	Dietary Approaches to Stop Hypertension
aHEI	alternate Healthy Eating Index
OR	odds ratio
aOR	confounder-adjusted odds ratio
IFPS II	Infant Feeding Practices Study II
FDA	Food and Drug Administration
CDC	Centers for Disease Control and Prevention
WIC	Special Supplemental Nutrition Program for Women, Infants, and Children
DHQ	Diet History Questionnaire
NCI	National Cancer Institute
HEI	Healthy Eating Index
SD	standard deviation
ANOVA	analysis of variance
CI	confidence interval
USD	U.S. dollar
NHANES	National Health and Nutrition Examination Survey
SCFA	short-chain fatty acid
HbA1c	glycated hemoglobin A1c

## Appendix A

**Table A1:** Comparison of socio-demographic and pregnancy-related characteristics between the analytic and excluded samples.

	Analytic Sample (N = 1397)		Excluded Sample (N = 3505)		
Characteristics *	n (%)	Mean ± SD	n (%)	Mean ± SD	*p*-Value
**Age, years**		28.8 ± 5.6		27.9 ± 5.8	**<0.001**
**% of federal poverty level**		257.5 ± 189.0		243.9 ± 200.1	**0.025**
**Race/ethnicity**					
Non-Hispanic White	1161 (83.8)		2702 (80.2)		**0.016**
Non-Hispanic Black	67 (4.8)		233 (6.9)		
Hispanic	93 (6.7)		242 (7.2)		
Non-Hispanic Asian/Pacific Islander/other	65 (4.7)		191 (5.7)		
**Highest education level**					
1–8 years of grade school	4 (0.3)		14 (0.5)		**<0.001**
High school	271 (20.9)		767 (25.7)		
1–3 years of college	519 (40.0)		1238 (41.5)		
College graduate	383 (29.5)		707 (23.7)		
Postgraduate	120 (9.3)		255 (8.6)		
**College education**					
Did not attend college	275 (21.2)		781 (26.2)		**<0.001**
Attended college	1022 (78.8)		2200 (73.8)		
**Mothers’ employment status**					
Unemployed	496 (35.7)		1139 (32.7)		**0.046**
Employed	895 (64.3)		2347 (67.3)		
**Household size**					
1–2 people	358 (25.6)		953 (27.2)		**0.011**
3 people	527 (37.7)		1148 (32.8)		
4 people	282 (20.2)		774 (22.1)		
5+ people	230 (16.5)		630 (18.0)		
**Annual household income level**					
<USD 25,000	312 (22.3)		893 (25.5)		**0.009**
USD 25,000–<USD 40,000	314 (22.5)		843 (24.1)		
USD 40,000–<USD 60,000	317 (22.7)		784 (22.4)		
≥USD 60,000	454 (32.5)		985 (28.1)		
**WIC recipient status**					
Non-recipient	823 (58.9)		1838 (52.4)		**<0.001**
Recipient	574 (41.1)		1667 (47.6)		
**Region**					
New England	68 (4.9)		138 (3.9)		0.277
Middle Atlantic	165 (11.8)		454 (13.0)		
East North Central	281 (20.1)		702 (20.0)		
West North Central	136 (9.7)		292 (8.3)		
South Atlantic	230 (16.5)		621 (17.7)		
East South Central	79 (5.7)		222 (6.3)		
West South Central	151 (10.8)		401 (11.4)		
Mountain	136 (9.7)		290 (8.3)		
Pacific	151 (10.8)		385 (11.0)		
**Smoking during pregnancy**					
No	1240 (89.3)		3041 (87.4)		0.065
Yes	149 (10.7)		440 (12.6)		

SD, standard deviation. * For some characteristics, the sum of categories is less than the total due to missing data. Significant *p*-values (<0.05) are in bold.

**Table A2:** Socio-demographic and pregnancy-related characteristics of participants in the analytic sample by dried bean consumption levels.

	Among Mothers Who Never Consumed Dried Beans (N = 633)		Among Mothers Who Consumed Dried Beans 1 Time per Month (N = 214)		Among Mothers Who Consumed Dried Beans 2–3 Times per Month (N = 320)		Among Mothers Who Consumed Dried Beans 1 Time per Week or More (N = 229)		*p*-Value
Characteristics *	n (%)	Mean ± SD	n (%)	Mean ± SD	n (%)	Mean ± SD	n (%)	Mean ± SD	
**Age, years**		28.3 ± 5.5		28.7 ± 5.8		28.9 ± 5.4		30.2 ± 5.3	**<0.001**
**% of federal poverty level**		257.8 ± 193.4		248.1 ± 189.0		255.8 ± 189.7		268.6 ± 176.0	0.721
**Race/ethnicity**									**0.037**
Non-Hispanic White	540 (85.7)		175 (82.2)		271 (85.2)		174 (77.7)		
Non-Hispanic Black	33 (5.2)		11 (5.2)		15 (4.7)		8 (3.6)		
Hispanic	32 (5.1)		15 (7.0)		17 (5.4)		29 (13.0)		
Non-Hispanic Asian/Pacific Islander/other	25 (4.0)		12 (5.6)		15 (4.7)		13 (5.8)		
**Highest education level**									0.477
1–8 years of grade school	2 (0.3)		1 (0.5)		1 (0.3)		0 (0.0)		
High school	135 (22.8)		47 (23.9)		57 (19.1)		31 (14.8)		
1–3 years of college	242 (40.9)		72 (36.6)		126 (42.3)		79 (37.8)		
College graduate	163 (27.5)		61 (31.0)		85 (28.5)		74 (35.4)		
Postgraduate	50 (8.5)		16 (8.1)		29 (9.7)		25 (12.0)		
**College education**									0.091
Did not attend college	137 (23.1)		48 (24.4)		58 (19.5)		31 (14.8)		
Attended college	455 (76.9)		149 (75.6)		240 (80.5)		178 (85.2)		
**Mothers’ employment status**									0.502
Unemployed	217 (34.4)		83 (38.8)		114 (35.7)		82 (36.3)		
Employed	414 (65.6)		131 (61.2)		205 (64.3)		144 (63.7)		
**Household size**									0.925
1–2 people	163 (25.8)		54 (25.2)		86 (26.9)		55 (24.0)		
3 people	245 (38.7)		83 (38.8)		115 (35.9)		83 (36.2)		
4 people	132 (20.9)		39 (18.2)		62 (19.4)		49 (21.4)		
5+ people	93 (14.7)		38 (17.8)		57 (17.8)		42 (18.3)		
**Annual household income level**									0.300
<USD 25,000	149 (23.5)		55 (25.7)		70 (21.9)		37 (16.2)		
USD 25,000–<USD 40,000	129 (20.4)		49 (22.9)		79 (24.7)		57 (24.9)		
USD 40,000–<USD 60,000	150 (23.7)		42 (19.6)		73 (22.8)		52 (22.7)		
≥USD 60,000	205 (32.4)		68 (31.8)		98 (30.6)		83 (36.2)		
**WIC recipient status**									0.198
Non-recipient	373 (58.9)		115 (53.7)		189 (59.1)		146 (63.8)		
Recipient	260 (41.1)		99 (46.3)		131 (40.9)		83 (36.2)		
**Region**									**<0.001**
New England	37 (5.9)		11 (5.1)		10 (3.1)		10 (4.4)		
Middle Atlantic	91 (14.4)		21 (9.8)		23 (7.2)		30 (13.1)		
East North Central	145 (22.9)		37 (17.3)		69 (21.6)		30 (13.1)		
West North Central	76 (12.0)		23 (10.8)		29 (9.1)		8 (3.5)		
South Atlantic	102 (16.1)		33 (15.4)		53 (16.6)		42 (18.3)		
East South Central	22 (3.5)		15 (7.0)		19 (5.9)		22 (9.6)		
West South Central	51 (8.1)		26 (12.2)		44 (13.8)		30 (13.1)		
Mountain	54 (8.5)		24 (11.2)		36 (11.3)		22 (9.6)		
Pacific	55 (8.7)		24 (11.2)		37 (11.6)		35 (15.3)		
**Smoking during pregnancy**									0.577
No	556 (88.4)		187 (87.8)		283 (89.3)		213 (93.0)		
Yes	73 (11.6)		26 (12.2)		34 (10.7)		16 (7.0)		

SD, standard deviation. * For some characteristics, the sum of categories is less than the total due to missing data. Significant *p*-values (<0.05) are in bold.

**Table A3:** Socio-demographic and pregnancy-related characteristics of participants in the analytic sample by chili consumption levels.

	Among Mothers Who Never Consumed Chili (N = 903)		Among Mothers Who Consumed Chili 1 Time per Month (N = 312)		Among Mothers Who Consumed Chili 2 or More Times per Month (N = 179)		*p*-Value
Characteristics *	n (%)	Mean ± SD	n (%)	Mean ± SD	n (%)	Mean ± SD	
**Age, years**		29.0 ± 5.4		28.4 ± 5.7		28.3 ± 5.8	0.118
**% of federal poverty level**		266.0 ± 198.0		244.5 ± 171.1		236.3 ± 169.7	0.062
**Race/ethnicity**							**0.038**
Non-Hispanic White	765 (85.0)		254 (83.0)		139 (78.5)		
Non-Hispanic Black	33 (3.7)		20 (6.5)		14 (7.9)		
Hispanic	63 (7.0)		20 (6.5)		10 (5.7)		
Non-Hispanic Asian/Pacific Islander/other	39 (4.3)		12 (3.9)		14 (7.9)		
**Highest education level**							**<0.001**
1–8 years of grade school	0 (0.0)		2 (0.7)		2 (1.3)		
High school	175 (20.4)		58 (21.1)		37 (23.3)		
1–3 years of college	328 (38.1)		126 (45.8)		64 (40.3)		
College graduate	267 (31.1)		72 (26.2)		44 (27.7)		
Postgraduate	90 (10.5)		17 (6.2)		12 (7.6)		
**College education**							**<0.001**
Did not attend college	175 (20.4)		60 (21.8)		39 (24.5)		
Attended college	685 (79.7)		215 (78.2)		120 (75.5)		
**Mothers’ employment status**							0.702
Unemployed	317 (35.3)		109 (35.2)		70 (39.1)		
Employed	582 (64.7)		201 (64.8)		109 (60.9)		
**Household size**							0.220
1–2 people	227 (25.1)		81 (26.0)		49 (27.4)		
3 people	363 (40.2)		113 (36.2)		50 (27.9)		
4 people	175 (19.4)		63 (20.2)		43 (24.0)		
5+ people	138 (15.3)		55 (17.6)		37 (20.7)		
**Annual household income level**							0.531
<USD 25,000	200 (22.2)		65 (20.8)		46 (25.7)		
USD 25,000–<USD 40,000	195 (21.6)		80 (25.6)		39 (21.8)		
USD 40,000–<USD 60,000	201 (22.3)		76 (24.4)		40 (22.4)		
≥USD 60,000	307 (34.0)		91 (29.2)		54 (30.2)		
**WIC recipient status**							0.234
Non-recipient	549 (60.8)		176 (56.4)		96 (53.6)		
Recipient	354 (39.2)		136 (43.6)		83 (46.4)		
**Region**							**<0.001**
New England	52 (5.8)		10 (3.2)		6 (3.4)		
Middle Atlantic	126 (14.0)		28 (9.0)		11 (6.2)		
East North Central	186 (20.6)		64 (20.5)		31 (17.3)		
West North Central	96 (10.6)		26 (8.3)		13 (7.3)		
South Atlantic	158 (17.5)		43 (13.8)		28 (15.6)		
East South Central	34 (3.8)		25 (8.0)		20 (11.2)		
West South Central	88 (9.8)		39 (12.5)		23 (12.9)		
Mountain	78 (8.6)		36 (11.5)		22 (12.3)		
Pacific	85 (9.4)		41 (13.1)		25 (14.0)		
**Smoking during pregnancy**							0.846
No	802 (89.5)		275 (88.4)		160 (89.4)		
Yes	94 (10.5)		36 (11.6)		19 (10.6)		

SD, standard deviation. * For some characteristics, the sum of categories is less than the total due to missing data. Significant *p*-values (<0.05) are in bold.

**Table A4:** Socio-demographic and pregnancy-related characteristics of participants in the analytic sample by bean soup consumption levels.

	Among Mothers Who Never Consumed Bean Soup (N = 1140)		Among Mothers Who Consumed Bean Soup 1 Time per Month (N = 211)		Among Mothers Who Consumed Bean Soup 2 or More Times per Month (N = 41)		*p*-Value
Characteristics *	n (%)	Mean ± SD	n (%)	Mean ± SD	n (%)	Mean ± SD	
**Age, years**		28.7 ± 5.5		29.3 ± 5.7		30.3 ± 6.1	0.075
**% of federal poverty level**		258.6 ± 187.9		261.4 ± 201.1		220.2 ± 162.6	0.425
**Race/ethnicity**							**0.001**
Non-Hispanic White	948 (83.8)		179 (84.8)		31 (77.5)		
Non-Hispanic Black	58 (5.1)		7 (3.3)		2 (5.0)		
Hispanic	71 (6.3)		16 (7.6)		5 (12.5)		
Non-Hispanic Asian/Pacific Islander/other	54 (4.8)		9 (4.3)		2 (5.0)		
**Highest education level**							0.244
1–8 years of grade school	3 (0.3)		1 (0.5)		0 (0.0)		
High school	231 (21.7)		34 (17.5)		4 (11.8)		
1–3 years of college	430 (40.4)		73 (37.6)		14 (41.2)		
College graduate	310 (29.1)		62 (32.0)		11 (32.4)		
Postgraduate	91 (8.5)		24 (12.4)		5 (14.7)		
**College education**							**0.043**
Did not attend college	234 (22.0)		35 (18.0)		4 (11.8)		
Attended college	831 (78.0)		159 (82.0)		30 (88.2)		
**Mothers’ employment status**							**<0.001**
Unemployed	401 (35.2)		79 (37.6)		15 (38.5)		
Employed	737 (64.8)		131 (62.4)		24 (61.5)		
**Household size**							0.102
1–2 people	292 (25.6)		53 (25.1)		13 (31.7)		
3 people	436 (38.3)		76 (36.0)		12 (29.3)		
4 people	240 (21.1)		35 (16.6)		7 (17.1)		
5+ people	172 (15.1)		47 (22.3)		9 (22.0)		
**Annual household income level**							0.313
<USD 25,000	251 (22.0)		46 (21.8)		14 (34.2)		
USD 25,000–<USD 40,000	257 (22.5)		46 (21.8)		8 (19.5)		
USD 40,000–<USD 60,000	253 (22.2)		56 (26.5)		7 (17.1)		
≥USD 60,000	379 (33.3)		63 (29.9)		12 (29.3)		
**WIC recipient status**							0.240
Non-recipient	676 (59.3)		125 (59.2)		21 (51.2)		
Recipient	464 (40.7)		86 (40.8)		20 (48.8)		
**Region**							0.116
New England	55 (4.8)		11 (5.2)		2 (4.9)		
Middle Atlantic	136 (11.9)		20 (9.5)		8 (19.5)		
East North Central	235 (20.6)		42 (19.9)		3 (7.3)		
West North Central	121 (10.6)		13 (6.2)		2 (4.9)		
South Atlantic	193 (16.9)		27 (12.8)		9 (22.0)		
East South Central	57 (5.0)		20 (9.5)		2 (4.9)		
West South Central	123 (10.8)		23 (10.9)		5 (12.2)		
Mountain	103 (9.0)		30 (14.2)		2 (4.9)		
Pacific	117 (10.3)		25 (11.9)		8 (19.5)		
**Smoking during pregnancy**							0.911
No	1007 (88.9)		191 (91.0)		38 (92.7)		
Yes	126 (11.1)		19 (9.1)		3 (7.3)		

SD, standard deviation. * For some characteristics, the sum of categories is less than the total due to missing data. Significant *p*-values (<0.05) are in bold.
